# Microneedle patches – the future of drug delivery and vaccination?

**DOI:** 10.3762/bjnano.14.40

**Published:** 2023-04-14

**Authors:** Zahra Faraji Rad, Philip D Prewett, Graham J Davies

**Affiliations:** 1 School of Mechanical and Electrical Engineering, University of Southern Queensland, Springfield Central, QLD 4300, Australia; 2 Department of Mechanical Engineering, University of Birmingham, Birmingham B15 2TT, United Kingdom; 3 Oxacus Ltd, Dorchester-on-Thames, OX10 7HN, United Kingdom; 4 Faculty of Engineering, UNSW Sydney, Sydney, NSW 2052, Australia; 5 College of Engineering & Physical Sciences, School of Engineering, University of Birmingham, Birmingham, B15 2TT, United Kingdom

Hypodermic needles and cannulas have been in clinical use since the 17th century. The first bevelled metal hypodermic needles were introduced by Francis Reed in 1844, followed by the syringe and needle combination, due to Alexander Wood, in 1853. Needles for a single intravenous dose (IV push) or bolus normally use a fixed intravenous hollow needle. Hypodermic syringe injections are, of course, ubiquitous in modern medicine for drug therapy and vaccination, where oral administration is either not desirable or not possible. Delivery may be intravenous, intramuscular or percutaneous. Hypodermic needles of various dimensions are also used to extract venous blood for diagnostic tests. Other tests, such as blood glucose monitoring in diabetics [1], release blood by a pinprick from the capillaries immediately beneath the skin.

Microneedles (MNs), typically less than 1 mm long, are a late 20th century development with significant promise for the above applications [2]. Recent research has also revealed a growing interest in diagnostic testing using the interstitial fluid (ISF) transdermally extracted, for example using suction devices [3], and there is increasing recognition by doctors and biomedical scientists of the potential role of the ISF in medical diagnostics. Microneedles provide shallow transdermal access to the ISF and are an excellent match to these and other developments when integrated into arrays on a substrate to form a patch.

The possibility of inexpensive mass-manufactured MN patches for drug delivery, vaccination, and diagnostic testing is a highly desirable clinical objective for the above reasons. They have the added advantage of being short, not stimulating nerve endings, therefore painless in use and attractive for patients prone to needle phobia. Indeed, patient self-administration is an option.

The commercial availability at scale of inexpensive disposable MN patches has been heralded for some years as a paradigm shift. Patches without MNs are already used for analgesic and anti-inflammatory treatments, nicotine addiction, and hormone replacement therapies. However, the combination of drug-loaded patches with MNs is still in its infancy, and MN patch diagnostic systems barely appear on the research landscape. Microneedle vaccination patches are closer to clinical acceptance and have enormous promise, given the demand for high volume, low cost, rapidly deployable vaccination in response to pandemics like COVID-19 [4], and companies dedicated to MN patch vaccination are already established. The key to the future of MN patches is the development and commercial availability of reliable, inexpensive, and biocompatible MNs with regulatory approval for clinical use.

The earliest MNs were made by adapting microfabrication technology originally developed for the microelectronics industry; they were inevitably made from silicon. Since then, silicon MNs have been largely abandoned in favour of polymer versions because of their superior mechanical properties, biocompatibility, ease of manufacture, and ultimate scalability [5–6]. Polymer MNs tailored to penetrate the skin and provide access to blood capillaries or the ISF have been the subject of a rapidly growing number of research publications over the last decade, and the trend continues [7]. Several key issues discussed in these papers are also considered in this thematic issue [8–9]. Recent progress may be broadly categorised as MN design, fabrication, skin penetration studies, and applications, ranging from drug delivery and vaccination to diagnostics. The first two of these have received most attention, but considerable work is still to be done in all categories – more in vivo studies for example. In the meantime, progress towards large-scale manufacture of moulded polymer MNs is progressing at pace, with the aid of new advanced 3D mould fabrication tools [10].

This special edition provides a snapshot of current research into MNs and their applications. It focuses on vaccination and drug delivery, but there is growing evidence of future potential in diagnostics and even in plant science [11]. If the thematic issue helps to inform existing researchers and to encourage others to join them, as editors, we will meet our objectives, the ultimate goal being the acceptance and availability of regulatory-approved MN patches for a wide range of clinical and biomedical applications.

We are grateful to the authors for their excellent contributions to this thematic issue; we are very aware of the demands on their time. We also thank the Beilstein-Institut editorial team for their highly professional support and the editorial board for approving this thematic issue.

Zahra Faraji Rad, Philip Prewett, and Graham Davies

Springfield, Birmingham and Sydney, March 2023
